# School-based group intervention in attention and executive functions: Intervention response and moderators

**DOI:** 10.3389/fpsyg.2022.975856

**Published:** 2022-09-15

**Authors:** Mika Paananen, Henrik Husberg, Heli Katajamäki, Tuija Aro

**Affiliations:** ^1^Niilo Mäki Institute, Jyväskylä, Finland; ^2^Department of Psychology, University of Jyväskylä, Jyväskylä, Finland; ^3^Department of Education, University of Helsinki, Helsinki, Finland

**Keywords:** executive functions, intervention, treatment efficacy, moderators, conduct problems, cognitive abilities

## Abstract

**Objective:**

This study investigated the effects of a school-based skill-training intervention in attention control and executive functions for pupils with hyperactivity-impulsivity (H-I) and cognitive control (CC) deficits. The main aim was to examine whether the intervention differently influenced H-I and CC, and whether cognitive abilities or conduct problems moderated response to the intervention.

**Method:**

Elementary school pupils from 41 schools participated the study and were divided into an intervention group (*n* = 71) and a waitlist control group (*n* = 77). Intervention outcomes were assessed with an inventory assessing executive function difficulties (including H-I and CC) completed by classroom teachers.

**Results:**

Significant intervention effects and positive changes were detected in CC but not in H-I. Significant intervention effects were found mainly among pupils with low levels of conduct problems.

**Conclusions:**

The results suggest that a skill-training intervention has specific positive effects on CC, but conduct problems may diminish response to intervention.

## School-based group intervention in attention and executive functions: Intervention response and moderators

Executive functions (EF) enable us to control our thoughts and actions (Barkley, 2012) and are thus pivotal for adapting to our environment. Sufficient EF enable self-regulation of behavior through more advanced functions, such as planning, adapting and evaluating one's behavior (Nigg, 2017). These abilities are especially needed in daily activities in a school setting where they are employed both in academic situations, and more broadly, in situations, including social interactions (Boekaerts, 1999). On a basic level, difficulties in EF compromise children's abilities to inhibit responses and distracting stimuli, to maintain or focus attention to update information in working memory, and to flexibly switch the focus of attention (Miyake et al., 2000; Pfiffner et al., 2006; Nigg, 2017). Difficulties with EF are fairly common in school-age children and are often associated with the diagnosis of attention deficit-hyperactivity disorder (ADHD; Swanson, 2003). However, even relatively mild EF difficulties can influence daily functioning, impede academic work and social interactions (Loe and Feldman, 2007; Moore et al., 2018) and on-task behavior (Sonuga-Barke, 2002). Inattention, in particular, may cause persistent impairments in daily functioning and result in completing fewer tasks and in less practice in learning situations, which may eventually lower academic achievement (Pfiffner et al., 2014). Thus, effective interventions accessible at school are needed to prevent adverse outcomes of EF deficits in learning.

Studies focusing specifically on children with ADHD have found the three nominal subtypes of deficits [predominantly inattentive, predominantly hyperactive-impulsive (H-I), and combined type; American Psychiatric Association, 2013] to be relatively unstable over time, and an alternative dimensional model using the amount of inattention and H-I symptoms has been suggested (Willcutt et al., 2012). Executive function deficits seem to be mostly related to the inattentive type of ADHD but to a lesser extent to H-I symptoms (Martel et al., 2007). Due to the continuum-like nature of EF deficits, children without a formal diagnosis are also known to be at risk of poor academic outcomes and are likely to need support at school (Loe and Feldman, 2007; Lahey and Willcutt, 2010). It has also been shown that especially symptoms of inattention and poor ability to flexibly adjust behavior in learning situations seem to be deleterious to academic success (Aro et al., 2005; DuPaul and Volpe, 2009; Sayal et al., 2015). Therefore, interventions should especially target attention, and focus on enhancing skills that enable efficient learning in classroom settings. These skills comprise maintenance of attention, adjusting behavior according to task demands as well as active maintenance of goal-oriented behavior, and they can be seen as the main components of cognitive control (CC; Nigg, 2017).

School-based interventions targeting attention and EF (e.g., Bikic et al., 2017; Evans et al., 2018) can focus on improving skills required in school learning, which has been shown to enhance the generalization of the effects to learning and on-task behavior (Moore et al., 2018). However, as the variation in outcomes of such interventions is large (Evans et al., 2018; Moore et al., 2018), more knowledge is needed about the effects of school-based interventions and, in particular, about the factors moderating the response to them. This study examined the effect of a comprehensive school-based intervention (*Maltti;* Paananen et al., 2011) in multi-sited and real school learning contexts. The intervention was provided for elementary pupils with attention and EF difficulties. It comprised behavioral, cognitive, and skills training elements, and it aimed to improve participants' on-task behavior in learning situations. In this study, we first investigated whether the intervention influenced the changes CC needed in on-task situations in schools. Second, we investigated whether pupils' pre-intervention characteristics (i.e., cognitive skills and conduct problems) moderated intervention outcomes. More precise knowledge of the intervention outcomes and of factors related to response can inform us about what effects can be expected and observed in classroom settings, and to whom this type of support should be offered.

### Training interventions

Previously, behavioral interventions (parent training, classroom management and combinations) focusing on behavioral problems have been shown to be particularly effective (Evans et al., 2014; Fabiano et al., 2015), but children with deficits in EF may also need support for executive and attentional skills (Pfiffner et al., 2014). Focusing only on problem behavior—that is, impulsivity, inefficiency and task avoidance—may lead to ignoring possible skill deficits that might be partly producing the observable behavior (Barkley, 1997). Therefore, skills training interventions targeting context-specific skills (inhibition, planning and strategies) and aiming to mitigate impairments produced by EF deficits are needed (Hinshaw et al., 2015).

Evidence suggests that the effects of school-based skill-focused interventions emerge if school-related skills are practiced in the intervention (Langberg et al., 2011, 2018; Abikoff et al., 2013; Evans et al., 2014; Re et al., 2015). Effects have been reported on cognitive skills, such as EF, planning and organization skills (Evans et al., 2014, 2016, 2018; Pfiffner et al., 2014; Langberg et al., 2018). The effects on teacher-rated inattention and H-I in classroom have varied (Evans et al., 2018). In their intervention study, Pfiffner et al. (2014) detected improvements in teacher-reported inattentive symptoms, whereas Miranda et al. (2013) found effects on both H-I and inattentive symptoms. Furthermore, the observed intervention effects on inattention and H-I may vary depending on whether the informant is a teacher or a parent (Moore et al., 2018). These previous studies have mainly focused on behavior related to ADHD, and cognitive aspects have gained less attention. In their intervention study, Capodieci et al. (2019) found that a group-based skill-focused intervention improved participants' control of attention, inhibition, and performance in reasoning tasks. This suggests that skill-centered interventions may benefit CC. Because better knowledge of the specific effects of school-based training interventions targeting EF skills needed in school could guide further intervention development in school settings, we analyzed whether school-based skills training had effects specifically on CC or also on H-I.

### Moderators of intervention effects

Even though school-based interventions on EF can be effective, variations in outcomes are common (Evans et al., 2018; Moore et al., 2018). A better understanding of the moderators of intervention response could help identify individuals who could potentially benefit from certain intervention types in school settings. Existing evidence suggests that among children with diagnosed ADHD, the severity of ADHD symptoms moderates the intervention response (Hinshaw, 2007; Moore et al., 2018), but less is known about the moderating effects of pre-intervention cognitive skills. We were especially interested in cognitive skills as moderators because problems with EF are known to be associated with cognitive deficits. Especially, language skills and working memory are proposed as cognitive prerequisites of EF (Barkley, 1997; Vygotsky, 2012) and deficits in these (Bruce et al., 2006; Friedman et al., 2022) as well as in visuo-spatial processing (Cardillo et al., 2020) are known to co-occur with attention problems. However, only few studies have addressed effects of cognitive abilities on intervention outcomes. Langberg et al. (2013) showed that cognitive ability did not moderate the response to training intervention, whereas the Multimodal Treatment of ADHD study (MTA; a multisite study evaluating behavior therapy, medications, and the combination of the two) found that low IQ in association with severe inattention and H-I symptoms and parental depression was related to a worse response (Hinshaw, 2007). Due to a lack of evidence and the strong association between cognitive abilities and intervention outcomes, we addressed the influence of verbal and visuo-constructive skills and working memory on the intervention response.

Although EF deficits often co-occur with behavioral problems (e.g., Barkley et al., 2006; Miyake and Friedman, 2012; Danforth et al., 2016), there is uncertainty about how they influence the intervention response. The MTA study (Hinshaw, 2007; Hinshaw et al., 2015) showed that co-occurring conduct or oppositional defiant disorders (ODD) did not moderate intervention outcomes among children with diagnosed ADHD. On the other hand, because Langberg et al. (2018) suggested that the effectiveness of homework and organizational skill interventions might vary more strongly among children with severe behavioral problems (H-I and ODD symptoms), the present study examined the extent to which conduct problem symptoms influenced intervention responses.

### Present study

We investigated the outcomes of a manualised school-based skills training intervention (*Maltti;* Paananen et al., 2011) on teacher-rated on-task and learning behaviors in the classroom. The group intervention was implemented in the Finnish elementary school context as part of conventional special educational support. First, we tested the intervention's effects on CC and H-I. Given that the intervention aimed to improve inhibition in on-task situations and task completion strategies, and based on previous studies (Pfiffner et al., 2014; Capodieci et al., 2019), we hypothesized that positive changes would be found in CC, but not in H-I. Second, based on theories on the development of EF (Barkley, 1997; Vygotsky, 2012; Cardillo et al., 2020), we also tested whether pre-intervention language skills, visuo-spatial skills, (i.e., WISC-IV Vocabulary and Block Design scores) and working memory (WISC-IV WM Index) moderated intervention response. As there is no previous knowledge of the cognitive moderators of school-based training interventions, we did not set a hypothesis. Third, we investigated whether the pre-intervention level of conduct problem symptoms moderated the intervention outcomes. As previous findings suggest that conduct problems do not play an obvious role as moderators (Hinshaw et al., 2015), we hypothesized that they would not affect the intervention outcomes.

## Methods

**Figure 1:**
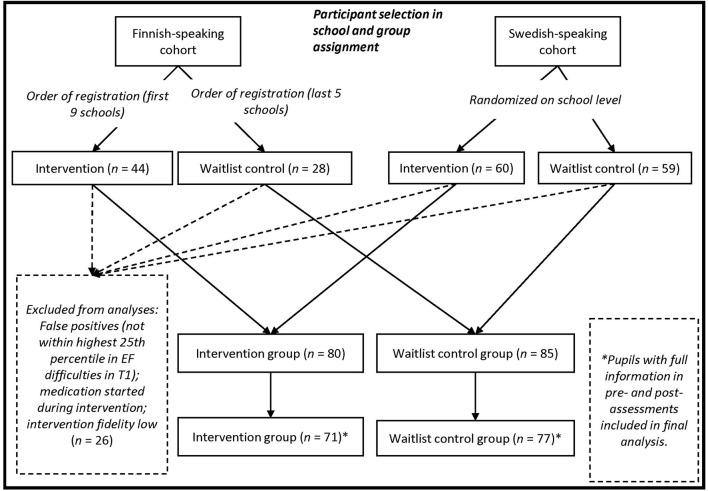
Flow chart for participant selection.

The study was conducted in Finnish primary schools over two different cohorts during the years 2013-2017. The first cohort consisted of Finnish-speaking schools and the second of Swedish-speaking schools (both are official languages in Finland). School personnel from the participating schools were provided a handbook with detailed instructions and intervention materials, and three to four training sessions were provided by the researchers. Training attendees were divided into intervention or waitlist group based on order of registration (first cohort) or by randomizing at the school level (second cohort). Altogether, 25 experimental intervention groups, with a total of 104 pupils, were formed. Because the objective was to investigate the Maltti-intervention implemented-as-designed (see Nelson et al., 2012), only intervention groups with high fidelity were included in the main analyses (19 groups with a total of 80 pupils; see Figure 1), but we also tested how intervention fidelity influenced intervention outcomes. The interventions were delivered on average one session per week excepting holidays during the school year (August-June in Finland), starting in October or November and ending in May.

### Participants

The first inclusion criterion for the intervention was presented during the first training session to intervention providers as follows: “*Children having symptoms of inattention, hyperactivity, and/or executive difficulties to such a degree that they impaired children's academic progress*.” The classroom teachers were asked to observe and select candidates for participation based on this criterion. The final decisions regarding students' participation in the intervention were made by school personnel, usually by a multi-professional group, and taking parents' views into consideration. This is in accordance with typical special education procedures in Finland, where diagnosis is not required for special educational support, but decisions are based on in-school evaluations (Björn et al., 2016). In addition to the EF deficits observed, the overall EF deficit symptoms must be in the top 25^th^ percentile based on the ATTEX EF deficits questionnaire filled in by teachers. Written consent was obtained from parents for both intervention and study participation.

Altogether, 191 pupils were assigned to the intervention group (*n* = 104) or waitlist group (*n* = 87). The participants' first languages were Finnish (*n* = 71 [43 in intervention group], 37.2%), Swedish (*n* = 119 [60 in intervention group], 62.3%), and Russian (*n* = 1 [in intervention group]; 0.5 %). All participants followed the standard curriculum and were fluent in the instructional language. Pupils who started ADHD medication during the intervention period, pupils not within the top 25^th^ percentile regarding EF difficulties and children in intervention groups with low implementation fidelity were excluded from the analysis of intervention effect. Pupils with full information in pre- and post- assessments were included in the final analysis (Figure 1).

Pupils came from 41 different primary schools across districts in Western, South, Eastern, and Central Finland (intervention group: 17 (32.0%) urban, 8 (68.0%) sub-urban or rural; waitlist group: 9 (56.3%) urban, 7 (43.8%) sub-urban or rural). In Finland, differences in pupils' performances between schools are very small (Arffman et al., 2010; Mullis, 2017). Further, the national curriculum and the Basic Education Act stipulate common guidelines for teaching and the provision of support at schools across municipalities. Accordingly, we did not expect significant variations between pupils' schooling and abilities, but we did test intervention and waitlist groups' equivalency for the basic descriptive variables and the possible influence of school on intervention effects.

### Intervention providers

The intervention training for the first cohort of providers consisted of four six-hour sessions, one in the spring semester and one in the fall before the intervention period, and two during the school year when interventions were running. This training showed that it could be carried out in a shorter period of time, therefore, for the second cohort, training consisted of three six-hour sessions, one in the spring semester and one in the fall before the intervention period, and one when the interventions were running. No financial or otherwise incentives were offered to the providers.

In the first cohort, the intervention providers were recruited for the study from a cost-free Maltti-intervention (Paananen et al., 2011) training course targeted to school personnel (teachers, special education teachers, and school psychologists). The course was financed by the Finnish Board of Education. Attending the study was voluntary for the training course participants who were all novices in terms of the Maltti-programme before the training and the study. In the second cohort, intervention providers were recruited by directly contacting all Swedish-speaking elementary schools in Finland with students in grades 1–6, for whom we could find updated contact information. All participants who signed up and attended the training course were asked to participate in the study, and all agreed. The participants were all novices in terms of the Maltti-program. The training courses were provided by Finnish-Swedish non-profit foundations.

All the intervention providers worked in the schools where the intervention took place. Of the providers, 25 (55.6%) worked as special education teachers, 10 (22.2%) as general education teachers, 7 (15.6%) as school psychologists, and 3 (6.7%) as social workers. Teachers and psychologists hold a higher education master's degree. Social workers also hold either a master or bachelor's degree. Because the intervention was delivered within special education, none of the providers was expected to be the classroom teacher of the participating pupils. In the experimental group, three intervention groups (with 13 pupils) had one of their two providers as also their classroom teacher, and therefore, the same teachers also evaluated participants' behavior in classrooms.

### Intervention

The Maltti-programme (“Patience”; Paananen et al., 2011) is targeted at children aged 7 to 11 years who have difficulties in attention control and task execution in classroom learning situations due to attentional and EF difficulties. The program includes 20 detailed instructions and materials for 60 to 75 mins intervention sessions for groups of four to six children, conducted in school facilities (Table 1). Maltti consists of exercises and tasks emphasizing three aspects of on-task and learning behavior: (i) Inhibition and attention control, (ii) Action selection and planning, and (iii) Strategy-use. The program consists of three phases during which children practice (1) allocation of attention to relevant stimuli and verbalisation (sessions 1–5), (2) regulation of one's own behavior in task situations to inhibit prepotent or overlearned reactions and behaviors (sessions 6–8), and (3) working and task completion strategies (sessions 9–20). Maltti employs *behavioral methods* (i.e., clear, effective and positive worded instructions and contingency-based behavior management systems) to enhance participants' on-task skills; *cognitive and skill-training* (i.e., modeling, verbalisation, strategy training and suppression of automatic or overlearned responses) to enhance attentional control and prolonged time of information processing, and *stop and go signals* to prolong the information processing time and to support systematic working. All sessions are structured following the same schedule. The routines and rules are explicit and remain unchanged throughout the sessions. Pupils are given feedback and they earn tokens. The sessions end with a game or playful activity intended to be a pleasant conclusion of each session.

**Table 1:** Treatment features of the Maltti intervention.

	**Targets**	**Tasks, materials and methods**	**Required skills**	**Predetermined goals for earning tokens**
Phase 1 (sessions 1–6)	Allocation of attention to relevant stimuli and verbalisation of tasks	• Modeling and scaffolding • Tasks: visual and auditory attention tasks (e.g., visual searching tasks, auditory repetition task)	•Attention control (focus, sustaining) • Focusing on relevant information • Verbalisation and reflection of perceptions and thoughts	• Completion of task and verbalisation of solutions • Completion of task according instructions • Completion of task as instructed in a specific order
Phase 2 (sessions 6–8)	Regulation of one's own behavior in task situations to inhibit prepotent reactions and behaviors	• Stop and go signals (Stop and green light signs) showing time for planning and thinking and completing a task • Tasks: visual searching tasks (e.g., organizing card series), coding, visual problem solving (e.g., reasoning of the rules in visual-problem solving tasks)	• Task and problem definition • Inhibition of reactive behaviors • Control of actions	• Using Stop and go signal to complete tasks • Verbalisation of the solutions
Phase 3 (sessions 9–20)	Working and task completion strategies	• Modeling • Task completion strategy use: (a) problem definition, (b) planning, (c) completion of a task according the plan and (d) reviewing the task completion • Tasks: visual and logical problem-solving tasks, social problem-solving tasks, reading comprehension and mathematical verification and problem-solving tasks (strategy use in these tasks)	• Task and problem definition and planning • Sustained attention • Flexible adaptation of actions • Cooperative working with other pupils	• Completions of tasks according task completion strategy • Completions of tasks independently according task completion strategy • Verbalisation of the solutions • Cooperative planning and prosocial behaviors (working in pairs, helping other pupils)
**Structure**	**“Warm-up”**	**Practices**	**Feedback and tokens**	**Game or play practices**
	“How are you” round and review of previously learned skills and presentation of new materials and skills	Practices of required skills with materials and tasks	A token system; pupils earned stickers or points, and by collecting them, pupils received rewards at after earning an agreed number of tokens	Board games, card games or playing in a group; intended to be a pleasant ending for each session and to improve/maintain positive group cohesion

### Measures and assessments

#### Measures

Language skills were assessed using the Vocabulary and visuo-spatial skills were assessed with the Block Design subtests and working memory with the Working Memory Index (WMI) from the Wechsler Intelligence Scale for Children (WISC IV; Wechsler, 2010). Vocabulary and Block Design subtests were selected as they correlate highly with the respective indices (*r* = 0.86 Vocabulary with Verbal Comprehension Index and *r* = 0.77 Block Design with Perceptual Reasoning Index) of the WISC IV (Wechsler, 2010).

Symptoms of conduct problems were assessed with the teacher-completed Conduct Problems subscale of the Strengths and Difficulties Questionnaire (SDQ; Goodman, 1997). The score ranges from 0 to 10, with 0–2 points indicating average levels, 3 points slightly raised, 4 points high, and 5–10 points very high levels of conduct problems (Borg, 2013). The Cronbach's alpha in a Finnish cohort of 7- to 9-year-olds for the Conduct Problem scale ranged from 0.66 (for girls) to 0.73 (for boys). In the present sample, Cronbach's α was 0.71.

Overall EF symptoms, H-I and CC were measured using the teacher-completed ATTEX questionnaire (Klenberg et al., 2010). It comprises 55 items in 10 subscales: Distractibility, Impulsivity, Hyperactivity, Directing attention, Sustaining attention, Shifting attention, Initiative, Planning, Execution of action, and Evaluation. We used aggregated Total scores of all items and aggregated subscales for H-I (subscales 1–3, max. 40) and for CC (subscales 4–10, max. 70). The ATTEX has a high internal consistency (Total score α = 0.98; Klenberg et al., 2010). In the present study, high consistency was found for the Total score (α = 0.94; 55 items), H-I (α = 0.93; 20 items) and CC (α = 0.93; 35 items). As ATTEX items tap difficulties, a decrease in score is a positive result.

Social validity was measured in the second cohort with a questionnaire consisting of five statements (e.g., “*The intervention reduces the pupils' difficulties on-task behavior,”* “*The intervention is easy to implement”)*. The scores were on a Likert scale ranging from 1 (totally disagree) to 6 (totally agree). Cronbach's α for the scale was 0.85.

#### Assessments

The pre-intervention WISC-IV tests were completed by psychologists (either research group member or school psychologist) in schools during the school hours. Pre-intervention ATTEX-questionnaires were completed by teachers in the autumn before the intervention, post-intervention in the following spring, and follow-up in the following autumn, one month after the beginning of the school year. Parents reported ADHD diagnoses and medications before and after the intervention period. In the second cohort, social validity assessments were completed by intervention providers at the end of the intervention periods for both intervention and waitlist groups.

### Intervention fidelity

Intervention providers were given an intervention manual with detailed instructions for delivery. Fidelity was assessed with a checklist and interviews. The criteria for fidelity evaluations followed the structure of each intervention session. Providers fulfilled the criteria-checklist after each session to determine whether the intended topics (i.e., what's-up round, modeling, practices, feedback, and game/plays session) were covered. They were interviewed at the end of the intervention to evaluate and confirm the given fidelity information. In cases in which less than 80% of planned topics were covered during the intervention period, the group was excluded from the analyses.

### Statistical analyses

Interventions with high fidelity were delivered in two different language environments (Finnish and Swedish), in 19 different schools; therefore, the possible influences of language environment and school on intervention effects and the necessity of two-level or nested analysis were tested before answering the research questions. Preliminary analyses with one-way ANOVA were used to compare the groups at baseline in age, grade, ATTEX total score, H-I, CC, and conduct problem symptoms, cognitive skills, mothers' education, and proportion of ADHD diagnoses and medication use. Preliminary analysis also included the investigation of missing data. To test the influence of intervention fidelity on the intervention effect within the experimental group, univariate ANOVA was used setting intervention fidelity as a between-subject factor, and the ATTEX total gain score (from pre- to post-intervention) was set as the dependent variable. The average score of the social validity measure is reported.

To answer the research questions, mixed-model ANOVA was used to analyse changes in outcome measures (i) between pre- and post-intervention and (ii) between pre-intervention and follow-up assessments. In the models, the ATTEX scores at three time points (pre-intervention, post-intervention, and follow-up) were entered as within-subject factors and the group as a between-subject factor. Group × moderator interaction analyses were conducted to determine how moderators influenced the intervention outcomes. In case a group × moderator interaction was statistically significant or very close to statistical significance, additional analyses were conducted to determine how moderators influence the intervention outcomes. The moderator analyses (levels of cognitive abilities and conduct problem severity) were performed using the Johnson-Neyman method and Process Macro 3.4. (SPSS extension; Hayes, 2018) which allows to analyse a range of pre-intervention values in which groups differ in their intervention gain relative to values of the moderator values. The ATTEX pre-intervention and post-intervention assessment time point scores were set as the within-subject factors and the group × moderator as an interaction term. Pre-intervention ATTEX Total score was entered as a covariate to improve the accuracy of the model (Langberg et al., 2018).

## Results

### Results of the preliminary analysis

Comparison between the intervention and waitlist group participants did not reveal statistically significant differences in age, grade level, ATTEX scores, conduct problem symptoms, WISC-IV scores, or mothers' education (Table 2). The number of children with an ADHD diagnosis or with ADHD medication did not differ between the two groups. Language environment {[*F*_(1, 69)_] = 2.64, *p* = 0.172} or school {[*F*_(16, 53)_] = 1.44, *p* = 0.16} had no significant effect on the ATTEX gain score; thus, a single-level model could be used in the main analysis.

**Table 2:** Comparison of the baseline descriptive values between the intervention and waitlist groups.

**Group**			
	**Intervention**	**Waitlist control**	
	**group**	**group**	
**Variables**	**M (SD)**	**M (SD)**	**Significance of the mean difference**
***N***	**71**	**77**	
Age (years)	9.31 (1.11)	9.67 (1.28)	ns.
Grade level	3.07 (1.13)	3.32 (1.21)	ns.
ATTEX total score	59.39 (19.69)	59.68 (17.76)	ns.
H-I score	21.01 (10.36)	21.59 (8.63)	ns.
CC score	38.38 (13.08)	38.04 (13.53)	ns.
VoSs	7.61 (2.61)	7.35 (2.61)^a^	ns.
BDSs	8.59 (3.22)	8.81 (3.16)^b^	ns.
WMI	15.93 (5.17)	16.26 (4.85)^b^	ns.
SDQ CP score	2.16 (2.00)^c^	2.81 (2.14)^a^	ns.
Education mother	3.48 (1.23)^d^	3.33 (1.16)^e^	ns.
	**Percentage**		**Comparison of the proportions**
ADHD diagnose	11.3	9.1	ns.
ADHD medication	8.5	5.2	ns.

H-I, Hyperactivity-Impulsivity; CC, Cognitive control; VoSs, Vocabulary standard score; BDSs, Block design standard score; WMI, Working memory Index; CP score, Conduct problem score; Only pupils with full information in ATTEX pre- and post-assessments included.

a n = 75,

b n = 74,

c n = 70,

d n = 61,

e n = 55.

Missing data affecting pre- and post-assessments were mainly due to missing teacher ratings. Analysis of the randomness of missing of data using Little's CAR showed that it was completely at random (χ^2^ = 41.19, *p* = 0.066). There were no statistically significant differences in the outcome or moderator variables as measured at T1 between pupils missing data from any timepoints compared to pupils with complete data except for age, where pupils with missing data were slightly older {[*F*_(1, 162)_] = 12.219, *p* < 0.001}.

Overall, 6 intervention groups (19 participants) out of 25 had lower than 80 % fidelity rate. Fidelity influenced the ATTEX Total gain score {pre- vs. post-test; [*F*_(1, 90)_] = 14.97, *p* < 0.001, η_p_^2^ = 0.14; pre- vs. follow-up [*F*_(1, 89)_] = 16.43, *p* < 0.001, η_p_^2^ = 0.16}. The pupils in the low-fidelity intervention groups had, on average, more EF deficit symptoms in the post-assessments compared to the pre-intervention assessments (*M* = 6.55, *SD* = 16.12) and even more symptoms in the follow-up than in the post-assessments (*M* = 12.50, *SD* = 17.64). These six groups were excluded from the following analysis. In the second cohort social validity assessments were completed by 38 intervention providers (intervention, and waitlist after their intervention period). The results of the social validity assessments indicated high social validity, (*M* = 4.29*, SD* = 0.76).

### Intervention outcomes and moderation analysis

The analyses revealed that the intervention group improved more in CC compared to the waitlist group during the intervention {[*F*_(1, 146)_] = 5.15, *p* = 0.025, η_p_^2^ = 0.03; Table 3}. Although the achieved gain endured in the intervention group, comparison of pre- and follow-up phases between groups did not show a significant difference in change in CC. Comparisons of H-I between the different time points were not statistically significant (Table 3).

**Table 3:** ATTEX scores at pre-, post-, and follow-up assessments.

	**Intervention group**			**Waitlist control group**		
	**(*****n*** = **71)**			**(*****n*** = **77)**		
	**Pre M (SD)**	**Post M (SD)**	**^a^Follow-up M (SD)**	**Pre M (SD)**	**Post M (SD)**	**^a^Follow-up M (SD)**
ATTEX score						
H-I	21.01 (10.36)	18.35 (10.50)	17.28 (10.07)	21.57 (8.69)	20.15 (9.11)	19.31 (9.45)
CC	38.38 (13.08)	32.90 (14.64)^*^	32.66 (15.84)	38.04 (13.53)	37.17 (12.89)^*^	34.55 (15.30)

H-I, Hyperactivity-impulsivity; CC, Cognitive control;

* statistically significant differences between profile groups at the p < 0.05 level between pre- and post-assessments.

a In the follow-up assessment, the number of participants was 70 in the intervention group and 69 in the waitlist group.

**Figure 2:**
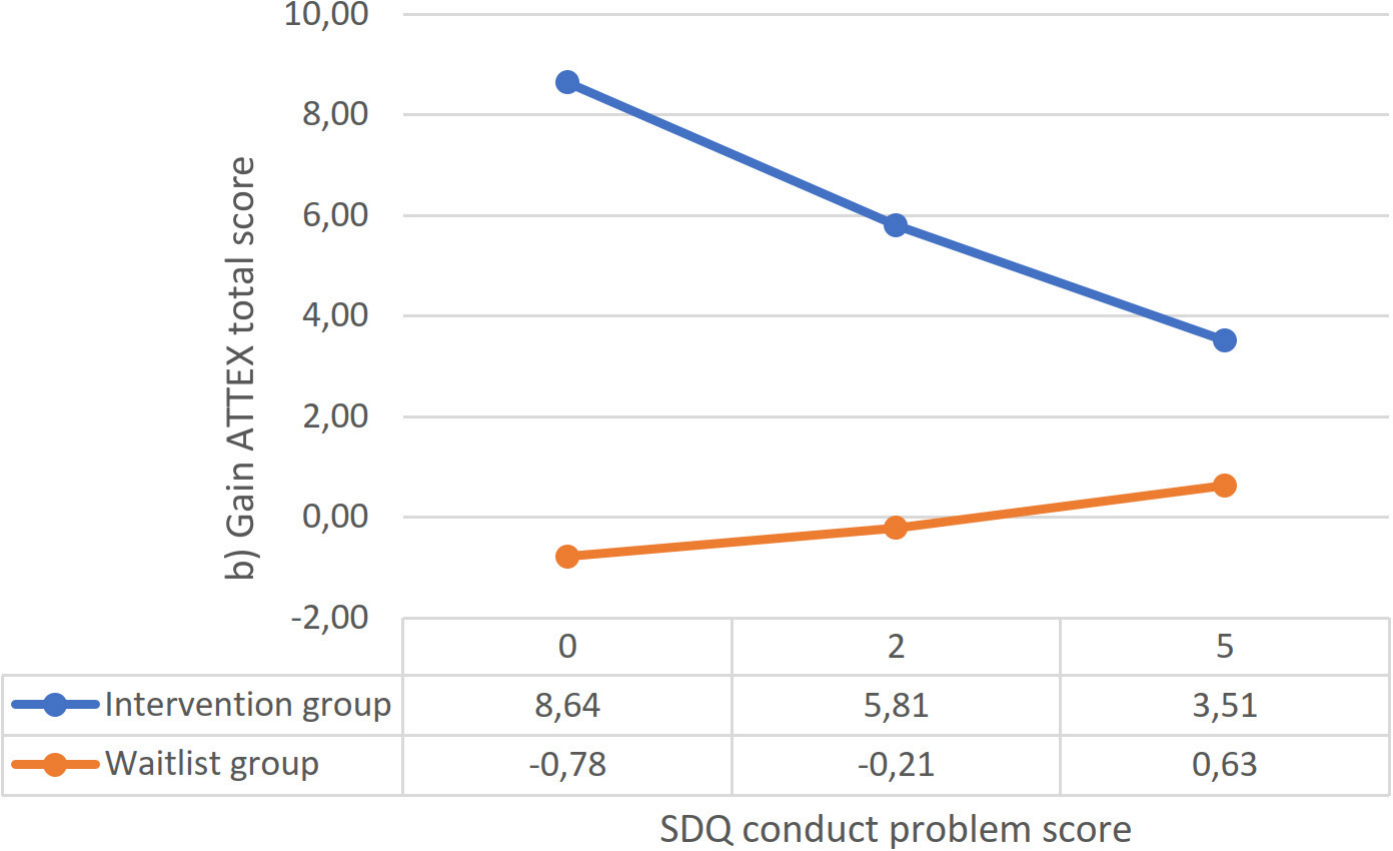
The linear connection between conduct problem symptoms and teacher-rated gain in the CC score (process analysis; Hayes, 2018). Statistically significant area of difference 0.00–3.20 for the ATTEX CC score on the SDQ conduct problem subscale.

A near statistically significant interaction effect of conduct problems and group on the ATTEX Total score and the CC {[*F*_(1, 140)_] = 3.42, *p* = 0.066} was found. The process moderator analysis showed that when the conduct problem score was low (i.e., ≤ 3), there was a significant relationship between group and the intervention gain; Maltti participants had significantly higher gains in the CC compared to the waitlist participants if their conduct problem scores were low (Figure 2). A total of 79% of the Maltti participants and 67% of the control participants were in this range of values of conduct problems. Conduct problems did not moderate the intervention effect on H-I. Pre-intervention language skills, visuo-spatial skills, or working memory scores did not have an effect on the intervention outcomes. As an additional analysis, we investigated the response between the groups among those whose conduct problem score was below three. The pairwise comparisons revealed that, among pupils with a low level of conduct problems, there was a larger imminent intervention effect in CC with medium effect size {[*F*_(1, 105)_] = 8.60, *p* = 004; η_p_^2^ = 0.08}.

## Discussion

The main purpose of the study was to explore the effect of a school-based intervention (Maltti) conducted as part of schools' special educational support on EF deficit symptoms observed in a classroom setting and, especially, to examine the moderators of the intervention response. The results revealed that the intervention supported the positive development of on-task skills and behaviors. More specifically, a significant intervention effect and positive change were detected in CC, but not in H-I, between pre- and post-assessments, which was in concordance with our expectations, as the intervention was targeted at skills that aim at improving attention and inhibition control in on-task situations and task completion strategies. Significant intervention effect was detected between pre- and post-assessments but not between pre- and follow-up assessments. Consistent with previous findings (e.g., Langberg et al., 2013), we found that pre-intervention language skills, visuo-spatial skills, or working memory did not moderate the intervention outcomes, whereas pre-intervention conduct problems moderated the intervention outcomes.

### Intervention effect

Altogether, the results indicated statistically significant effects and a functional relationship between the intervention and a change in behavior. Significant group differences in teacher-rated EF deficits and on-task behaviors were detected between pre- and post-assessments but not between pre-assessment and follow-up. Even so, problem behaviors continued to decrease until the follow-up measurement in the intervention group, demonstrating maintenance of the intervention effect. Although the intervention effect was statistically significant, it must be noted that the teacher-rated deficits showed, on group level, a 16% decrease. However, it could be argued that quite large and inherently socially valid changes are needed before being noticeable by the type of outcome measure used in this study. The changes in behavior resulting from the intervention were observed in the pupils' natural setting and associated with socially meaningful and important outcomes. In addition, social validity inventory showed that respondents agreed with statements that the intervention was effective and feasible.

The findings, showing intervention change in pupils' behavior in the classrooms, that is, outside the delivery setting, have pedagogical relevance, as they show pupils' ability to use the new skills in learning situations. One profound limitation of interventions for children with attention deficits has been the lack of generalization of the effects across different settings (Abikoff, 2009; Evans et al., 2018). Moreover, the gain was observed in CC, which is relevant for learning, and which was the target of the training during the intervention period; that is, allocating attention to relevant stimuli, regulation, and inhibition of prepotent reactions in on-task situations and strategy use and systematic working habits. The findings support the previous notion that training the specific skills needed in the classroom benefits children with deficits in EF (Abikoff et al., 2013; Pfiffner et al., 2014; Langberg et al., 2018). The Maltti intervention practices did not significantly diminish participants' H-I observable in classroom, suggesting that this type of cognitive training intervention does not have an influence on H-I, which is also in line with a previous study (Pfiffner et al., 2014).

It must be noted that the teacher-rated behavior change in the waitlist group was quite substantial between the post- and follow-up assessments. One reason for the positive change in behavior ratings in this group may be leaking of the Maltti intervention principles in the waitlist schools, which is possible in active and development-oriented schools. The training of the intervention providers of the waitlist school personnel started in the spring preceding the autumn in which they started their intervention period. The entire school year-long waiting period might have created an urge to start specific support in addition to “support as usual” for the pupils in the waitlist group.

### Moderator effects

Cognitive skills (i.e., language and visuo-spatial skills, working memory) did not moderate the response to the intervention, which is in accordance with a previous study by Langberg et al. (2013). This suggests that the materials and principles of the Maltti intervention enhanced the basic elements of on-task behaviors that are not related to or dependent on the assessed cognitive skills. Although the finding is encouraging, it should also be noted that the variation in cognitive skills was rather small, which may have diminished the power of the analysis. Thus, more research is needed on the effects of cognitive skills as moderators on intervention response, also outside skill training.

Conduct problems did influence the intervention outcomes; moderator analysis showed that in the intervention group, improvements were demonstrated mainly among pupils with low levels (average or slightly raised) of conduct problems. Our results indicated that school-based training interventions that focus on on-task skills do not necessarily benefit pupils with higher levels (high or very high) of conduct problem symptoms, which is in line with Langberg et al. (2018). There are several possible explanations for this result. Pupils with conduct problems may be either more resistant to behavior change, or defiant behavior and disobedience or oppositionality prevent active participation in group activities and practices, thus resulting in poorer development of on-task skills (McClelland et al., 2000). Further, generalization of the practiced skills may be weaker among pupils with conduct problems resulting from unchanged and negative interaction between teacher and pupil or pupil's tendency to stick to maladaptive and disruptive behavior. It is also plausible that the pupils with conduct problems had other types of distress not considered in this study, perhaps related to home or parenting. One prominent line of research and intervention development could therefore be the integration of parenting support and skill training for these pupils (see e.g., Leijten et al., 2020).

### Intervention fidelity

Previous research has shown that successful implementation of behavioral interventions and good implementation fidelity improve the intervention outcomes of evidence-based practices (Monzalve and Horner, 2020). Similarly, in the present study, the fidelity of the intervention implementation influenced intervention responses. An unanticipated finding was that low intervention fidelity intervention resulted in negative effects on pupils' on-task behaviors, which were detected even in the follow-up phase. It is likely that even not attending the intervention could have been more beneficial for these pupils, than attending the intervention group with poor fidelity. This raises intriguing questions on why and how low fidelity causes negative intervention effects, or what causes low fidelity. Pre-intervention data revealed that levels of pupils' attention deficit or conduct problem symptoms were not higher in groups with low fidelity; actually, levels of symptoms, both in attention deficit and conduct problems, were lower in the low fidelity intervention groups. Based on these data, we can infer that low fidelity was not associated with particularly difficult pre-intervention problem behaviors. Conducted fidelity interviews revealed that low fidelity may be associated, instead, with maladaptive adult-pupil interaction. It is possible that communication in the groups did not boost positive task orientation, and that intervention providers may have paid attention to negative behavior instead of task orientation and positive feedback, thereby triggering oppositional behavior in the pupils. This process could be understood from the point of view of the therapeutic alliance, as it has been shown that the formation and maintenance of a good child–therapist alliance has the potential to aid in achieving positive psychotherapy outcomes among adolescents (for a meta-analysis, see Karver et al., 2018). If communication between adults and pupils has been negatively toned, at worst some pupils may have interpreted the intervention as a punishment or felt that the adult is not “for me and with me.” This supplementary information raises important questions about the integrity of the interventions in the school context, and the topic needs to be further studied.

### Limitations

The study was conducted in a naturalistic setting that exposed it to some limitations. Full randomization of participants was not possible, and the study was conducted with a quasi-experimental design. Previously, some authors have strongly argued that unblinded assessments and the proximity of the assessor cause biased data (e.g., Sonuga-Barke et al., 2013). Teachers who rated pupils' behavior in the classroom setting were aware of the pupils' intervention condition and of the general goal of the intervention. However, there are several reasons to believe that the positive changes in behaviors reflected generalization of the practiced skills into the classroom settings rather than the expectation effect of the classroom teachers. The classroom teachers (except for three teachers out of 45 intervention providers who were involved in intervention delivery) did not receive any training component, nor were they explicitly familiarized with intervention ingredients or specific skills that were practiced in the intervention group sessions. Furthermore, intervention providers or teachers were not taught any methods to support the generalization of practiced skills into classroom settings. In addition, teacher rated evaluations reflected the level of intervention fidelity that teachers were not aware of. Although blinding the assessments for stakeholders in a naturalistic setting is difficult, a multi-informant method and systematic observations should be used in the future to diminish the possible biases in data. To control for the possible leak of training to the waitlist group, we recommend that future studies use successive multiple observation-based measurement points (systematic observations or direct behavior ratings; Christ et al., 2009). Such an assessment could reveal whether anticipation or the start of intervention has immediate effects on interpretations of children's behavior, or whether the intervention generates gradual change in behaviors as is expected in training interventions.

### Conclusions and future directions

The results of this study indicated that the school-based comprehensive group intervention combining behavioral, cognitive, and skills training methods positively influenced behavioral manifestations of CC in the classroom setting but did not influence H-I. The results also revealed that the intervention was more effective for pupils with fewer conduct problem symptoms. Given that the intervention response was minor among pupils with more severe conduct problems, we recommend other intervention methods, particularly those targeting conduct problems for these pupils (see Kazdin, 2018). To obtain greater improvements in both H-I and conduct problems, training interventions should be integrated with behavioral interventions focusing on behavioral control and acting-out behavior in the classroom (Pfiffner et al., 2006; Karhu et al., 2018; Capodieci et al., 2019). Accordingly, for children without attention and EF problems but with a primary need for H-I behavior support, behavioral management interventions should be preferred instead of, or before, training interventions.

A strength of this study was that it showed positive results from intervention that was conducted in ecological and multi-sited contexts with a low threshold for participation, that is, pupils were eligible for participation based on teacher identification of need for support. Along with better accessibility, interventions implemented with integrity in the school context are cost-effective compared to treatments outside school hours (Pfiffner et al., 2014). The study shows that even a relatively short training for school personnel may be enough to obtain competence to conduct a manualized group-based intervention that benefit pupils' EF. It must be noted that although the present results reflected positive intervention effects for pupils in the intervention group, especially for those with low levels of conduct problems, the downside was that poor intervention fidelity resulted in worsening on-task behavior in the classroom setting. This finding underlines the importance of the integrity of the intervention when trying to achieve positive changes in behavior and learning in a special educational setting. Further research should be undertaken to investigate the intervention fidelity and integrity, pupil-teacher interactions, and methods that enable generalization of special educational support to classroom settings.

As noted above the intervention had limited effects, especially on children with H-I and those with conduct problems. Therefore, in the future the implementation of individualized support plans alongside skill training may enable extending the intervention efforts to the classrooms (e.g., using functional behavior assessment-based approaches; March and Horner, 2002). Further, the use of multiple measurement points could assist in identifying for whom and in which conditions the intervention is beneficial and could also offer the possibility of modifying the intervention to improve its effect, especially for pupils not benefiting from it. Better knowledge of the factors influencing the successful implementation of the intervention programme would help to better integrate the intervention into the school context and to better offer support to children with EF deficits symptoms.

## Data availability statement

The raw data supporting the conclusions of this article will be made available by the authors, without undue reservation.

## Ethics statement

The studies involving human participants were reviewed and approved by Ethical Committee of University of Jyväskylä. Written informed consent to participate in this study was provided by the participants' legal guardian/next of kin.

## Author contributions

MP had a contribution to research design, implementation processes of the intervention, data collection, data analysis, and writing the article. HH and HP had a contribution to implementation processes of the intervention, data collection, data analysis, and writing the article. TA had a contribution to research design, implementation processes of the intervention and writing the article, and she was in role as a supervisor of the research group. All authors contributed to the article and approved the submitted version.

## Funding

This study was funded by the Academy of Finland (nos. 264415 and 264344 for 2013-2015), Svenska Kulturfondet Stiftelsen (nos. 160026 for 2015-2019), Brita Maria Renluns Minne (nos. 20-1663 for 2015-2019), and Svenska Folkskolans Vänner (nos. SFV-0204 for 2015-2019).

## Conflict of interest

The authors declare that the research was conducted in the absence of any commercial or financial relationships that could be construed as a potential conflict of interest.

## Publisher's note

All claims expressed in this article are solely those of the authors and do not necessarily represent those of their affiliated organizations, or those of the publisher, the editors and the reviewers. Any product that may be evaluated in this article, or claim that may be made by its manufacturer, is not guaranteed or endorsed by the publisher.

## References

[B1] Abikoff H. (2009). ADHD psychosocial treatments: generalization reconsidered. J. Attent. Disord. 13, 207–210. 10.1177/1087054709333385 19553560

[B2] Abikoff H. Gallagher R. Wells K. Murray D. Huang L. Lu F.. (2013). Remediating organizational functioning in children with ADHD: Immediate and long-term effects from a randomized controlled trial. J. Consult. Clinic. Psychol. 81, 113–128. 10.1037/a0029648 22889336 PMC3549033

[B3] American Psychiatric Association. (2013). Diagnostic and statistical manual of mental disorders (5th ed.). 10.1176/appi.books.97808904255968723190

[B4] Arffman I. Välijärvi J. Linnakyl,ä P. (2010). Finnish basic education: when equity and excellence meet, in Equity and Excellence in Education (London: Routledge), 202–226.

[B5] Aro T. Semrud-Clikeman M. Lapveteläinen A.-M. Lyytinen H. (2005). Developmental underpinnings of the association of ADHD and its subtypes to neuropsychological and academic weaknesses, in Attention Deficit Hyperactivity Disorder: From Genes to Patients, eds D. Gozal and D. Molfese (London: The Humana Press), 293–316.

[B6] Barkley R. (1997). Behavioral inhibition, sustained attention, and executive functions: Con-structing a unifying theory of ADHD. Psychologic Bull. 121, 65–94. 10.1037/0033-2909.121.1.65 9000892

[B7] Barkley R. Fischer M. Smallish L. Fletcher K. (2006). Young adult outcome of hyperactive children: adaptive functioning in major life activities. J. Am. Acad. Child Adolesc. Psychiatr. 45, 192–202. 10.1097/01.chi.0000189134.97436.e2 16429090

[B8] Barkley R. A. (2012). Executive functions: What they are, how they work, and why they evolved. Guilford Press.

[B9] Bikic A. Reichow B. McCauley S. Ibrahim K. Sukhodolsky D. (2017). Meta-analysis of organizational skills interventions for children and adolescents with Attention-deficit/hyperactivity disorder. Clinic. Psychol. Rev. 52, 108–123. 10.1016/j.cpr.2016.12.004 28088557

[B10] Björn P. Aro M. Koponen T. Fuchs L. Fuchs D. (2016). The many faces of special education within RTI frameworks in the United States and Finland. Learn. Disabil. Q. 39, 58–66. 10.1177/0731948715594787

[B11] Boekaerts M. (1999). Self-regulated learning: where we are today. Int. J. Educ. Res. 31, 445–457. 10.1016/S0883-0355(99)00014-2

[B12] Borg A.-M. (2013). Strengths and Difficulties Questionnaire. Available online at: www.terveysportti.fi/apps/dtk/tmi/article/tmm00147/search/sdq. (accessed April 9, 2021).

[B13] Bruce B. Thernlund G. Nettelbladt U. (2006). ADHD and language impairment. Euro. Child Adolesc. Psychiatr. 15, 52–60. 10.1007/s00787-006-0508-9 16514510

[B14] Capodieci A. Re A. M. Fracca A. Borella E. Carretti B. (2019). The efficacy of a training that combines activities on working memory and metacognition: transfer and maintenance effects in children with ADHD and typical development. J. Clinic. Experiment. Neuropsychol. 41, 1074–1087. 10.1080/13803395.2019.1651827 31401917

[B15] Cardillo R. Vio C. Mammarella I. C. (2020). A comparison of local-global visuospatial processing in autism spectrum disorder, nonverbal learning disability, ADHD and typical development. Res. Develop. Disabil. 103, 103682. 10.1016/j.ridd.2020.103682 32442872

[B16] Christ T. Riley-Tillman T. Chafoules S. (2009). Foundation for the development and use of direct behavior rating (DBR) to assess and evaluate student behavior. Assess. Effect. Intervent. 34, 201–213. 10.1177/1534508409340390

[B17] Danforth J. Connor D. Doerfler L. (2016). The development of comorbid conduct problems in children with ADHD: an example of an integrative developmental psychopathology perspective. J. Attent. Disord. 20, 214–229. 10.1177/1087054713517546 24412971

[B18] DuPaul G. J. Volpe R. J. (2009). ADHD and learning disabilities: Research findings and clinical implications. Curr. Dev. Disord. Rep. 1, 152–155.

[B19] Evans S. Langberg J. Schultz B. Vaughn A. Altaye M. Marshall S.. (2016). Evaluation of a school-based treatment program for young adolescents with ADHD. J. Consult. Clinic. Psychol. 84, 15–30. 10.1037/ccp0000057 26501496

[B20] Evans S. Owens J. Bunford N. (2014). Evidence-based psychosocial treatments for children and adolescents with attention-deficit/hyperactivity disorder. J. Clinic. Child Adolesc. Psychol. 43, 527–551. 10.1080/15374416.2013.850700 24245813 PMC4025987

[B21] Evans S. Owens J. Wymbs B. Ray A. (2018). Evidence-based psychosocial treatments for children and adolescents with attention deficit/hyperactivity disorder. J. Clinic. Child Adolesc. Psychol. 47, 157–198. 10.1080/15374416.2017.1390757 29257898

[B22] Fabiano G. Schatz N. Aloe A. Chacko A. Chronis-Tuscano A. (2015). A systematic review of meta-analyses of psychosocial treatment for attention-deficit/hyperactivity disorder. Clinic. Child Fam. Psychol. Rev. 18, 77–97. 10.1007/s10567-015-0178-6 25691358 PMC4346344

[B23] Friedman L. M. Rapport M. D. Fabrikant-Abzug G. (2022). Consistently inconsistent working memory performance among children with ADHD: Evidence of response accuracy variability (RAV). J. Psychopathol. Behav. Assess. 2, 1–13. 10.1007/s10862-022-09967-7

[B24] Goodman R. (1997). The strengths and difficulties questionnaire: a research note. J. Child Psychol. Psychiatr. 38, 581–586. 10.1111/j.1469-7610.1997.tb01545.x 9255702

[B25] Hayes A. (2018). Introduction to mediation, moderation, and conditional process analysis: A regression-based approach (2nd edition). New York, NY: The Guilford Press.

[B26] Hinshaw S. (2007). Moderators and mediators of treatment outcome for youth with ADHD: understanding for whom and how interventions work. J. Pediatric Psychol. 32, 664–675. 10.1093/jpepsy/jsl055 17264086

[B27] Hinshaw S. Arnold L. the MTA Cooperative Group (2015). Attention-deficit hyperactivity disorder, multimodal treatment, and longitudinal outcome: evidence, paradox, and challenge. Wiley Interdisciplin. Rev. Cogn. Sci. 6, 39–52. 10.1002/wcs.1324 26262927 PMC4280855

[B28] Karhu A. Närhi V. Savolainen H. (2018). Inclusion of pupils with ADHD symptoms in mainstream classes with PBS. Int. J. Inclus. Educ. 22, 475–489. 10.1080/13603116.2017.1370741

[B29] Karver M. De Nadai A. Monahan M. Shirk S. (2018). Meta-analysis of the prospective relation between alliance and outcome in child and adolescent psychotherapy. Psychotherap. 55, 341. 10.1037/pst0000176 30335449

[B30] Kazdin A. (2018). Implementation and evaluation of treatments for children and adolescents with conduct problems: findings, challenges, and future directions. Psychotherap. Res. 28, 3–17. 10.1080/10503307.2016.1208374 27449266

[B31] Klenberg L. Jäms,ä S. Häyrinen T. Lahti-Knuuttila P. Korkman M. (2010). The attention and executive function rating inventory (ATTEX): Psychometric properties and clinical utility in diagnosing ADHD subtypes. Scand. J. Psychol. 51, 439–448. 10.1111/j.1467-9450.2010.00812.x 20338019

[B32] Lahey B. Willcutt E. (2010). Predictive validity of a continuous alternative to nominal subtypes of attention-deficit/hyperactivity disorder for DSM-V. J. Clinic. Child Adolesc. Psychol. 39, 761–775. 10.1080/15374416.2010.517173 21058124 PMC3056555

[B33] Langberg J. Becker S. Epstein J. Vaughn A. Girio-Herrera E. (2013). Predictors of response and mechanisms of change in an organizational skills intervention for students with ADHD. J. Child Fam. Stud. 22, 1000–1012. 10.1007/s10826-012-9662-5 24319323 PMC3848056

[B34] Langberg J. Dvorsky M. Molitor S. Bourchtein E. Eddy L. Smith Z.. (2018). Overcoming the research-to-practice gap: A randomized trial with two brief homework and organization interventions for students with ADHD as implemented by school mental health providers. J. Consult. Clinic. Psychol. 86, 39. 10.1037/ccp0000265 29172596

[B35] Langberg J. M. Epstein J. N. Girio-Herrera E. Becker S. P. Vaughn A. J. Altaye M. (2011). Materials organization, planning, and homework completion in middle-school students with ADHD: Impact on academic performance. Sch Ment Health 3, 93–101. 10.1007/s12310-011-9052-y 23577045 PMC3619433

[B36] Leijten P. Scott S. Landau S. Harris V. Mann J. Hutchings J.. (2020). Individual participant data meta-analysis: Impact of conduct problem severity, comorbid attention-deficit/hyperactivity disorder and emotional problems, and maternal depression on parenting program effects. J. Am. Acad. Child Adolesc. Psychiatr. 59, 933–943. 10.1016/j.jaac.2020.01.023 32084529

[B37] Loe I. Feldman H. (2007). Academic and educational outcomes of children with ADHD. J. Pediatric Psychol. 32, 643–654. 10.1093/jpepsy/jsl054 17569716

[B38] March R. Horner R. (2002). Feasibility and contributions of functional behavioral assessment in schools. J. Emot. Behav. Disord. 10, 158–170. 10.1177/10634266020100030401

[B39] Martel M. Nikolas M. Nigg J. T. (2007). Executive function in adolescents with ADHD. J Am Acad Child Adolesc Psychiatry. 46, 1437–1444. 10.1097/chi.0b013e31814c953 18049293

[B40] McClelland M. Morrison F. Holmes D. (2000). Children at risk for early academic problems: the role of learning-related social skills. Early Childhood Res. Q. 15, 307–329. 10.1016/S0885-2006(00)00069-7

[B41] Miranda A. Presentación M. J. Siegenthaler R. Jara P. (2013). Effects of a psychosocial intervention on the executive functioning in children with ADHD. J. Learn. Disabil. 46, 363–376. 10.1177/0022219411427349 22064952

[B42] Miyake A. Friedman N. P. (2012). The nature and organization of individual differences in executive functions: four general conclusions. Curr. Dir. Psychol. Sci. 21, 8–14. 10.1177/0963721411429458 22773897 PMC3388901

[B43] Miyake A. Friedman N. P. Emerson M. J. Witzki A. H. Howerter A. Wager T. D. (2000). The unity and diversity of executive functions and their contributions to complex “Frontal Lobe” tasks: a latent variable analysis. Cogn. Psychol. 41, 49–100. 10.1006/cogp.1999.0734 10945922

[B44] Monzalve M. Horner R. (2020). The impact of the contextual fit enhancement protocol on behavior support plan fidelity and student behavior. Behav. Disord. 20, 497. 10.1177/0198742920953497

[B45] Moore D. Russell A. Matthews J. Ford T. Rogers M. Ukoumunne O.. (2018). School-based interventions for attention-deficit/hyperactivity disorder: a systematic review with multiple synthesis methods. Rev. Educ. 6, 209–263. 10.1002/rev3.3149

[B59] Mullis I. V. S. Martin M. O. Foy P. Hooper M. (2017). PIRLS 2016 International Results in Reading. TIMSS & PIRLS International Study Center website. Available online at: http://timssandpirls.bc.edu/pirls2016/international-results/

[B46] Nelson M. Cordray D. Hulleman C. Darrow C. Sommer E. (2012). A procedure for assessing intervention fidelity in experiments testing educational and behavioral interventions. J. Behav. Health Serv. Res. 39, 374–396. 10.1007/s11414-012-9295-x 22935907

[B47] Nigg J. (2017). Annual Research Review: On the relations among self-regulation, self-control, executive functioning, effortful control, cognitive control, impulsivity, risk-taking, and inhibition for developmental psychopathology. J. Child Psychol. Psychiatr. 58, 361–383. 10.1111/jcpp.12675 28035675 PMC5367959

[B48] Paananen M. Heinonen J. Knoll J. Leppänen U. Närhi V. (2011). Kummi 8. Maltti – tarkkaavuuden ja toiminnanohjauksen ryhmäkuntoutus. [Maltti – Group based intervention of deficits in attention and executive functions]. Niilo Mäki Instituutti.

[B49] Pfiffner L. Barkley R. DuPaul G. (2006). Treatment of ADHD in school settings, in Attention-deficit Hyperactivity Disorder. A Handbook for Diagnosis and Treatment, ed Barkley, R. (London: The Guilford Press), 547–589.

[B50] Pfiffner L. Villodas M. Kaiser N. Rooney M. McBurnett K. (2014). Educational outcomes of a collaborative school–home behavioral intervention for ADHD. School Psychol. Q. 28, 25–36. 10.1037/spq0000016 23506023 PMC4091627

[B51] Re A. M. Capodieci A. Cornoldi C. (2015). Effect of training focused on executive functions (attention, inhibition, and working memory) in preschoolers exhibiting ADHD symptoms. Front. Psychol. 6, 1161. 10.3389/fpsyg.2015.01161 26300836 PMC4526792

[B52] Sayal K. Washbrook E. Propper C. (2015). Childhood behavior problems and academic outcomes in adolescence: longitudinal population-based study. J Am Acad Child Adolesc Psychiatry. 54, 360–368. 10.1016/j.jaac.2015.02.007 25901772

[B53] Sonuga-Barke E. (2002). Psychological heterogeneity in AD/HD—A dual pathway model of behaviour and cognition. Behav. Brain Res. 130, 29–36. 10.1016/S0166-4328(01)00432-6 11864715

[B54] Sonuga-Barke E. Brandeis D. Cortese S. Daley D. Ferrin M. Holtmann M.. (2013). Nonpharmacological interventions for ADHD: systematic review and meta-analyses of randomized controlled trials of dietary and psychological treatments. Am. J. Psychiatr. 170, 275–289. 10.1176/appi.ajp.2012.12070991 23360949

[B55] Swanson J. M. (2003). Role of executive function in ADHD. J. Clinic. Psychiatr. 64,35–39. 14658934

[B56] Vygotsky L. S. (2012). Thought and Language. New York, NY: MIT press.

[B57] Wechsler D. (2010). WISC-IV-Wechsler Intelligence Scale for Children. [Käsikirja II: Teoriatausta, standardointi ja tulkinta]. Psykologien Kustannus.

[B58] Willcutt E. Nigg J. Pennington B. Solanto M. Rohde L. Tannock R.. (2012). Validity of DSM-IV attention-deficit/hyperactivity disorder dimensions and subtypes. J. Abnorm. Psychol. 121, 991–1010. 10.1037/a0027347 22612200 PMC3622557

